# Subterranean *Deuteraphorura* Absolon, 1901, (Hexapoda, Collembola) of the Western Carpathians — Troglomorphy at the northern distributional limit in Europe

**DOI:** 10.1371/journal.pone.0226966

**Published:** 2020-01-15

**Authors:** Andrea Parimuchová, Martina Žurovcová, Vladimír Papáč, Ľubomír Kováč

**Affiliations:** 1 Department of Zoology, Institute of Biology and Ecology, Faculty of Science, P.J. Šafárik University, Košice, Slovakia; 2 Institute of Entomology, Biology Centre AS CR v.v.i., České Budějovice, Czech Republic; 3 State Nature Conservancy of the Slovak Republic, Slovak Caves Administration, Cave Care Department, Rimavská Sobota, Slovakia; Nanjing Agricultural University, CHINA

## Abstract

An integrative approach employing molecular, morphological and geographical data were applied to species delimitation among *Deuteraphorura* congeners occupying caves of the Western Carpathian Mts. A new species of *Deuteraphorura* from the Western Carpathians is described. ***D*. *muranensis* sp. nov**. belongs among species with 4 pso at the hind margin of the head and possesses highly troglomorphic features. It is conspicuous with its distinctly elongated claws and long, hair-like body chaetae. The status of the new species was confirmed by DNA barcoding based on the mitochondrial COI marker. Populations of *D*. *kratochvili* (Nosek, 1963), the most widespread species, were studied in detail. Both ABGD and PTP analyses brought results congruent with geography, i.e. the molecular and geographic distance of the populations were positively correlated. However, some molecular separation based on pairwise distance and the number of substitutions was indicated within two of the studied populations. Despite the indistinct morphological differences, the tested populations were well isolated both geographically and genetically, which indicates that each studied population may represent a cryptic species. The troglomorphy of cave Collembola at the northernmost border of the distribution of cave-adapted species in the Europe is discussed. It is clear that the level of troglomorphy is closely associated with conditions of the microhabitat occupied by the individual subterranean species. The results of our study enhance the importance of the Western Carpathians regarding the diversity pattern of obligate cave species in Europe.

## Introduction

In Europe, a high diversity of cave-adapted species has been observed in so-called “hot-spot” areas of the southern European mountains, with a decreasing trend towards northern regions, where only a few troglobionts have been documented [1, 2, 3]. The distribution of obligate cave species in the Western Carpathian Mts, i.e. beyond troglobiont-rich areas, points out the importance of these karst regions as the northernmost distributional range borders of terrestrial troglobionts [4].

Caves represent extreme environments with specific conditions that generate selection pressure on cave inhabitants and lead to the convergence of morphological traits [5]. Variation in these features, even in closely related cave species, is a function of microhabitat partitioning, distinct especially in aquatic environments [6]. Changes in morphology associated with cave life may affect several structures related to darkness, food scarcity, air temperature and the presence of wet clayey sediment. Such changes are manifested in body size, body slenderness, eyes, pigment, appendage length, foot complex and wing development as progressive (the development of systems not seen in surface-dwelling organisms) or regressive (loss of systems occurring in surface-dwelling organisms) adaptations [7, 8, 9]. Along with general troglomorphisms widespread across cave-adapted taxa (larger body size, reduced eyes and pigment, elongated appendages and claws), specific modifications appear in cave Collembola: shorter, thinner and pointed tenent hairs on tibiotarsi compared to their edaphic counterparts, reduction and basal shift of the inner teeth on the claw, and hypertrophy and/or multiplication of antennal sensilla [8, 10].

Subfamily Onychiurinae as typically edaphic Collembola, are unpigmented and eyeless forms in which other troglomorphisms rarely develop. The exceptions are the three troglobiotic onychiurids, namely *Ongulonychiurus colpus* (Thibaud & Massoud, 1986) discovered in the deep abysses of Picos d´Europa (Spain); a new *Ongulonychiurus* from a deep cave in the Biokovo Mts (Croatia) [11]; and *Troglaphorura gladiator* Vargovitsh, 2019 [12], recently described from the Snezhnaya Abyss in the Caucasus Mts (Georgia). All species are characteristic with highly troglomorphic traits.

The genus *Deuteraphorura* (Poduromorpha, Onychiuridae) includes 83 species [13], 49 of which have been described from caves. Along others, the number and distribution of pseudocelli and parapseudocelli (hardly visible soft cuticular structures) over the body, play an important role in morphological identification of species of the family Onychiuridae. However, most onychiurids are well-characterized by abovementioned features, high variability of morphological characteristics and frequently also their left-right asymmetry (e.g. body chaetotaxy, number of pseudocelli and parapseudocelli on the body segments) is often in some species of the genera *Protaphorura* and *Deuteraphorura*.

Integrative taxonomy is a synthetic approach combining evidence from independent data sources (e.g. morphology, DNA, ecology, geography) in order to reveal biological features relevant for species delimitation [14, 15]. This integrative approach has been successfully applied to collembolan taxa, where the colour pattern was previously the only helpful characteristic used in their identification, e.g. [16, 17, 18, 19]. Many studies have reported congruent results when a combination of morphological and molecular approaches was used in species delimitation, e.g. [20, 21, 22, 23, 24]. In contrast, low morphological but clear genetic differences [17, 25, 26, 27, 28], or clear morphological but low genetic differences [29], have also been documented in Collembola. In this regard, the subfamily Onychiurinae seems to represent a serious challenge due to the uniform colouration, similar ecology and habitat preferences of its representatives and the often unstable and/or asymmetric chaetotaxy and pseudocellar pattern over the body, especially in genera *Protaphorura and Deuteraphorura* (e.g. [30]).

We assumed that the divergence of cave *Deuteraphorura* may be higher than previously expected, including older phyletic relicts in the territory of the Western Carpathians.

This contribution aimed at 1) evaluating the congruence between morphological traits, DNA barcode and geographical data for species delimitation of cave populations of the genus *Deuteraphorura* occupying the Western Carpathian caves; 2) clarifying the species status of *Deuteraphorura kratochvili* (Nosek, 1963), the most widespread troglobiotic species of the genus in the study territory; and 3) describing the new remarkably troglomorphic species of the genus *Deuteraphorura* discovered in the Muránska planina Plateau karst area. The level of troglomorphy in subterranean representatives of the genus *Deuteraphorura* is discussed regarding their distribution over the northernmost boundary of the range of terrestrial troglobionts in Europe.

## Material and methods

Individuals of the genus *Deuteraphorura* were collected by hand in caves of the Western Carpathians, Slovakia (see futher text), on cave sediment, speleothems, bat guano, rotten wood and the surface of the water pools and immediately fixed in 96% ethyl alcohol. For morphological study, the specimens were separately mounted on permanent slides in Swann medium (Liquido de Swann) modified after Rusek [31] and studied through a phase-contrast Leica DM 2500 microscope equipped with DIL optics (differential interference contrast), a measuring eyepiece (micrometric ocular) and a drawing arm. The images were taken with an Olympus 5.1 megapixel camera and edited using Adobe Photoshop CS6. Chaetotaxy of the tibiotarsus is presented after Deharveng [32], and chaetotaxy of the labium after Fjellberg [33]. Claw length was measured along the internal side as the distance from the distal margin of the pretarsus to the top of claw. Claw width (lateral view) was measured as the width of the pretarsus in conjunction with tibiotarsus.

**Fig 1:**
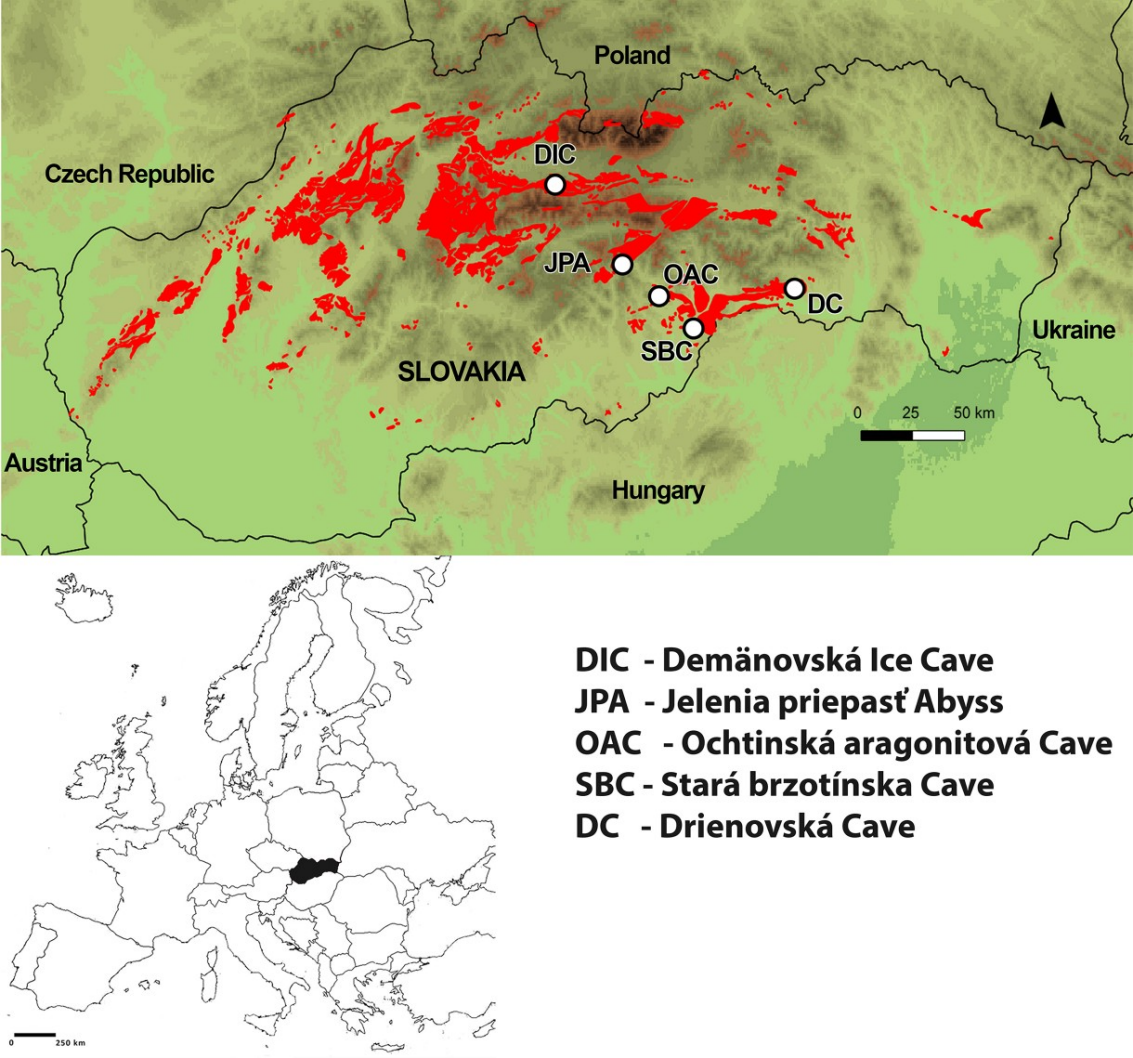
Location of the caves with the studied *Deuteraphorura* populations on the map (red spots represent karst areas).

Five populations of *Deuteraphorura* were included in the molecular analyses; four populations of *D*. *kratochvili* from the following caves: Demänovská Ice Cave (Low Tatras), Stará Brzotínska Cave (Slovak Karst), Drienovská Cave (Slovak Karst) and Ochtinská aragonitová Cave (Revúcka vrchovina Mts), and one population of *D*. *muranensis* sp.nov. from Jelenia priepasť Abyss (Muránska planina Plateau) (Fig 1). *Deuteraphorura kratochvili* populations were examined for the purpose of the species redescription [30]. In this study, populations were defined as individuals collected in separate caves. The selected caves are located in four orographic units in Slovakia, with a minimal air distance of 12 km between the closest caves (Stará Brzotínska Cave–Ochtinská aragonitová Cave) and a maximum air distance of 110 km between the most distant caves (Drienovská Cave–Demänovská Ice Cave).

The sampling of Collembola material was carried out with the permission of the Ministry of the Environment of the Slovak Republic (No. 2661/2017-6.3).

### Molecular data analysis

To prevent contamination, all DNA laboratory work was conducted under sterile conditions with the use of barrier tips. Total DNA was extracted using the Qiagen DNeasy Blood & Tissue Kit according to the modified manufacturer’s protocol (see [34]). Polymerase chain reaction (PCR) [35] was carried out using a 12.5 μL of reaction volume consisting of 1 μL of template DNA (not quantified), 10 × PCR Buffer (TopBio s.r.o., CZ), 12.5 mM of dNTP mix, 5 μM of each primer and 0.125 units of Taq polymerase (TopBio s.r.o., CZ) on a GenePro (Bioer Co., Ltd, China) thermal cycler. A fragment (670 bp) of the COI gene was amplified using the universal primers LCO1490 (5’-ggt caacaa atcataaagatattg g-3’) and HCO2198 (5’-taa act gggtga ccaaaaaat ca-3’; [36]). The thermal cycling conditions were as follows: 94°C for 1 min followed by 35 cycles (94°C for 30 sec, 47°C for 35 sec and 72°C for 50 sec), followed by 1 min 30 sec at 72°C. After verification via agarose gel electrophoresis, the reaction products were purified using Exo I/FastAP (Thermo Fisher Scientific Inc.). The sequencing of the purified products was performed using LCO1490 at the SEQme s.r.o. in Dobris, Czech Republic, by the Sanger method. In cases when the primer failed to produce high quality chromatograms, reverse primer sequencing was employed. Sequences were manually edited and trimmed of unreadable short stretches (ca 30 bp at the 5’ and 3’ ends) with Bioedit v.7 [37]. Since none of them contained stop codons or indels in ORF, all were considered to be true mitochondrial and not nuclear copies. All the sequences were verified as being consistent with Onychiuridae congeners using the GenBank BLASTn search (the Mega Blast algorithm with the default setting). Sequences were aligned with the MEGA 7.0.26 [38] software by the Muscle (Codons) algorithm using the Invertebrate Mitochondrial GeneCode and default parameters. Standard DNA barcoding distance analysis was conducted using the Tamura-3 parameter method [39] recommended by model selection. A neighbour-joining tree [40] with the Tamura-3 parameter method [39] was constructed, and the robustness of the tree nodes was assessed by bootstrap analysis with 1000 replications. *Deuteraphorura inermis* (Tullberg, 1869) (GenBank code KY231117.1) was used as the outgroup.

Both barcoding gap- and evolutionary models were applied for the COI marker. Automatic Barcode Gap Discovery (ABGD) clustered the sequences into candidate species based on pairwise distances by detecting barcoding gaps for the COI marker [41]. Prior intraspecific divergence varied from 0.001 (Pmin, a single nucleotide difference) to 0.14 (Pmax, threshold value) applied by Porco [42]. The relative gap width was set to 1, with 20 recursive steps, 40 bids for a graphic histogram of distances, a K2P model [43] for distance calculation and other parameters as the default.

The Poisson tree processes (PTP) model based on significant differences in the number of substitutions was performed using on-line software [44]. Identical sequences were removed to avoid incorrect likelihood calculations, and an unrooted NJ tree was constructed using the IQ-TREE web server with the default parameters (http://iqtree.cibiv.univie.ac.at/).

Correlation between the genetic and geographical distances of the populations was evaluated by means of the Mantel test (999 permutations) using the GenAlEx 6.5 program. Haplotype diversity (h) was calculated using DnaSP 5 [45]; subsequently, a haplotype network for the *Deuteraphorura* populations was constructed using the program Network 5.0.0.3 (website fluxus-engineering.com) (results not shown). The presence/absence of shared haplotypes was used to verify gene flow among the populations.

All the new sequences are available in GenBank (accession numbers for *D*. *muranensis*: MN727813 –MN727820 and for *D*. *kratochvili*: MN727821 –MN727844).

### Nomenclatural acts

The electronic edition of this article conforms to the requirements of the amended International Code of Zoological Nomenclature, and hence the new names contained herein are available under that Code from the electronic edition of this article. This published work, and the nomenclatural acts it contains, have been registered in ZooBank, the online registration system for the ICZN. The ZooBank LSIDs (Life Science Identifiers) can be resolved and the associated information viewed through any standard web browser by appending the LSID to the prefix “http://zoobank.org/”. The LSID for this publication is: urn:lsid:zoobank.org:act: AD211C26-DCCA-4042-A677-EBB1CBC528D6. The electronic edition of this work was published in a journal with an ISSN, and has been archived and is available from the following digital repositories: PubMed Central, LOCKSS.

## Results

Molecular analysis of the mitochondrial cytochrome oxidase I (COI) fragment was carried out in five *Deuteraphorura* populations, the morphology and chaetotaxy of which were examined for the purpose of *D*. *kratochvili* redescription [30].

Genetic diversity was calculated between and within species and populations, respectively (Table 1). In *D*. *kratochvili*, intraspecific divergence was 11%, while it approximated zero within particular populations, except for Demänovská Ice Cave, with 3.5%. A significant positive correlation between the geographical and genetic distances was confirmed by the Mantel test (R^2^ = 0.53; p = 0.001). In *D*.*muranensis* from Jelenia priepasť Abyss, intrapopulation divergence reached 4.8%.

**Table 1:** Genetic distances within and between *Deuteraphorura* species and populations (standard errors in italics; n/c–not calculable; see Fig 1 for cave abbreviations).

SPECIES							POPULATIONS								
		between			Within			between						within	
		1	2	3				outgroup	JPA	SBC	DIC	DC	OAC		
1	**outgroup**		*0*,*028*	*0*,*025*	n/c	*n/c*	**outgroup**		*0*,*028*	*0*,*029*	*0*,*026*	*0*,*026*	*0*,*027*	n/c	*n/c*
2	***D*.*muranensis***	0,247		*0*,*015*	0,048	*0*,*008*	**JPA**	0,247		*0*,*019*	*0*,*017*	*0*,*018*	*0*,*013*	0,048	*0*,*008*
3	***D*.*kratochvili***	0,232	0,130		0,110	*0*,*012*	**SBC**	0,251	0,147		*0*,*019*	*0*,*016*	*0*,*018*	0,002	*0*,*001*
							**DIC**	0,223	0,134	0,144		*0*,*020*	*0*,*018*	0,035	*0*,*007*
							**DC**	0,217	0,149	0,119	0,155		*0*,*019*	0,000	*0*,*000*
							**OAC**	0,237	0,090	0,132	0,133	0,145		0,001	*0*,*001*

Among 32 *D*. *kratochvili* sequences 13 haplotypes were detected, and none of them was shared among the populations. Each population was represented by 2–4 haplotypes, with the exception of Drienovská Cave, with only one haplotype.

**Fig 2:**
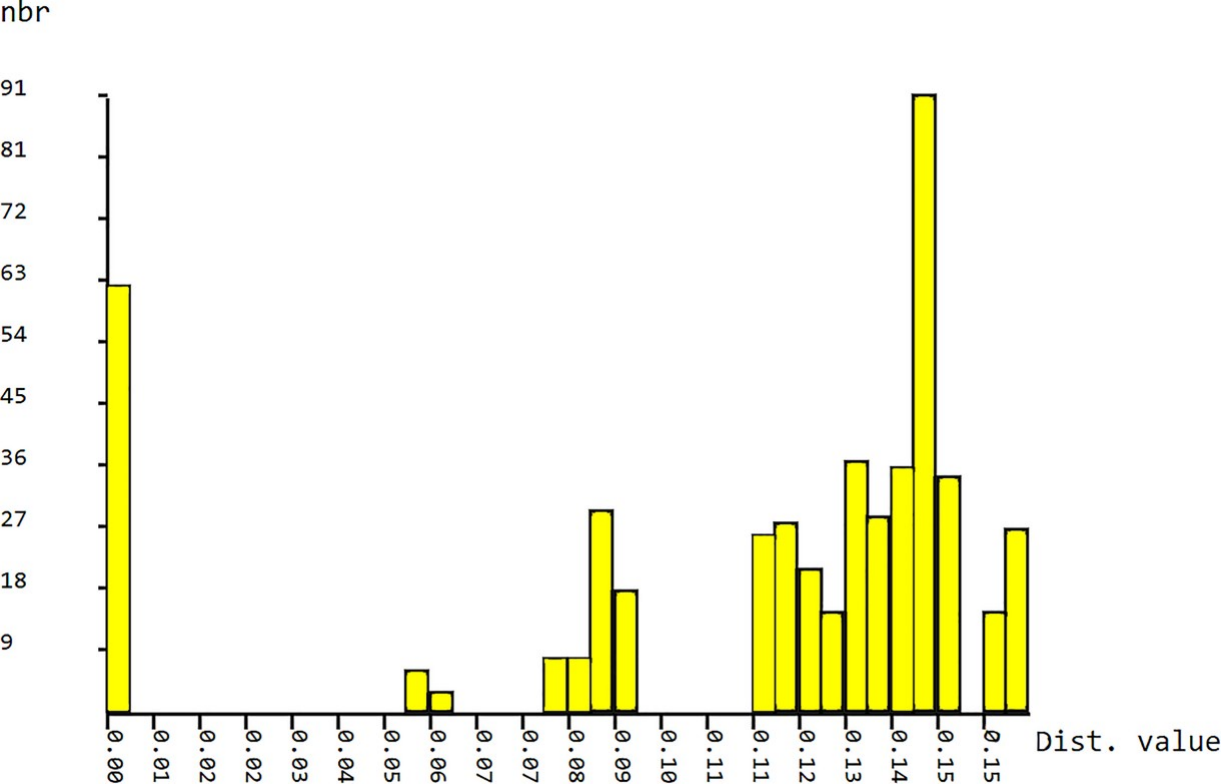
Histogram of genetic distances of *Deuteraphorura* with barcoding gaps.

ABGD detected prior intraspecific divergences (P) between 0.001 and 0.049, while 10 and 7 MOTUs were recognized in the initial and recursive partitions, respectively. Barcoding gaps at K2P distance were observed between 0.01–0.055, 0.065–0.075 and 0.095–0.11(Fig 2). Initial and recursive partitions were congruent at about P = 0.03, while the number of MOTUs was 7. ABGD species delimitation corresponded to particular populations; however, populations from the Jelenia priepasť Abyss and Demänovská Ice Cave were further divided into two partitions each.

**Fig 3:**
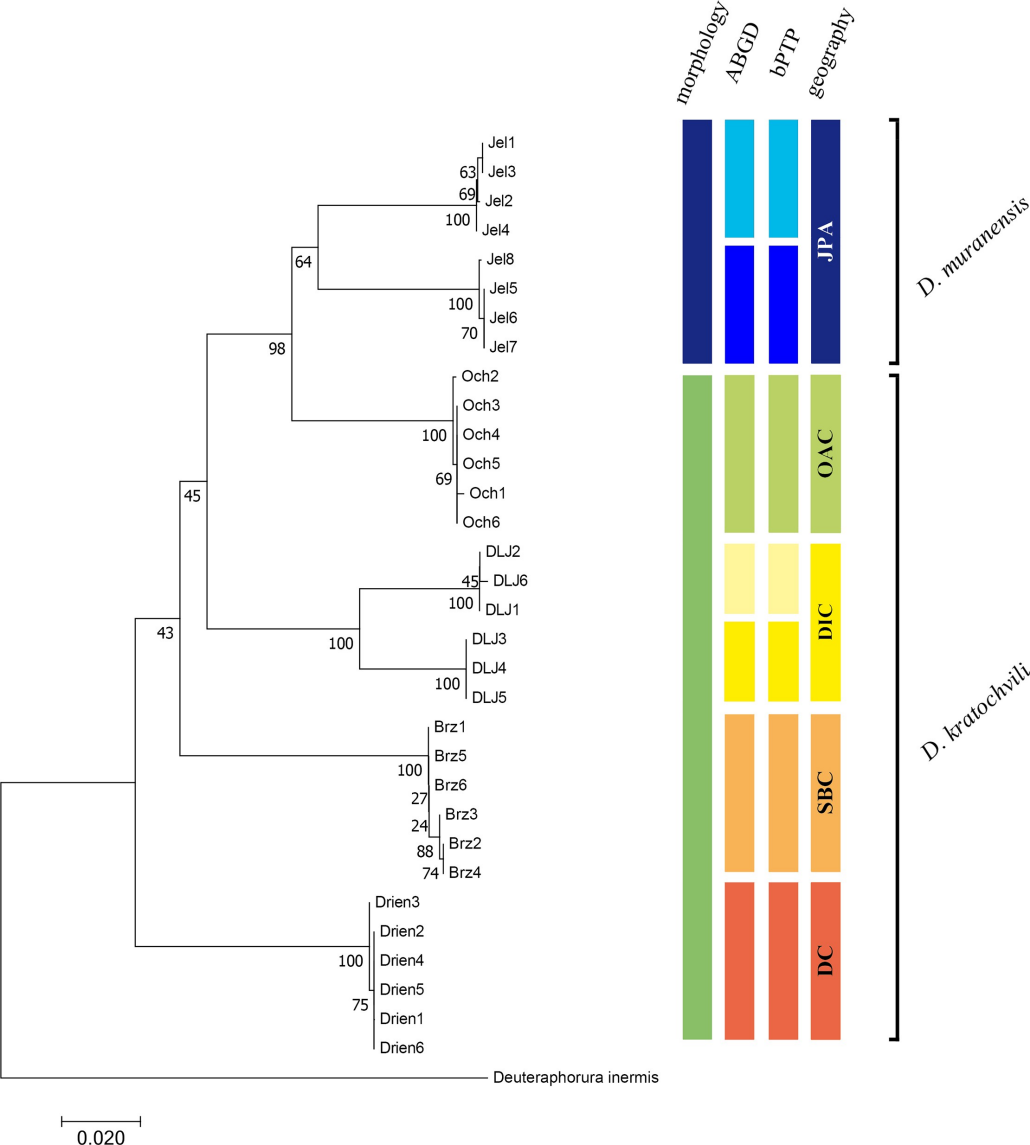
Consensus of different species delimitation approaches on an NJ-tree. Bootstrap values are indicated on the branches. Colour stripes represent populations.

PTP delimitation based on haplotype sequences identified 7 species/MOTUs (support 0.88–1.0), thus fitting the results of the AGBD analysis. Species delimitation approaches were summarized on an NJ-tree (Fig 3).

### Species description

#### Taxonomy

Family **Onychiuridae** Lubbock, 1867

Subfamily **Onychiurinae** Börner, 1901

Genus ***Deuteraphorura*** Absolon, 1901

**Diagnosis**: Postantennal organ with numerous compound vesicles. Sensory clubs in antenna III smooth with ribs. Bases of antenna well marked. Posterior pseudocelli on the head present. Furca reduced to finely granulated area with 2+2 chaetae posteriorly arranged in one row. Chaeta d0 on the head present. Anal spines absent. Distal verticil of chaetae on tibiotarsi with 9 chaetae. MVO usually present on Abd.II-III.

**Fig 4:**
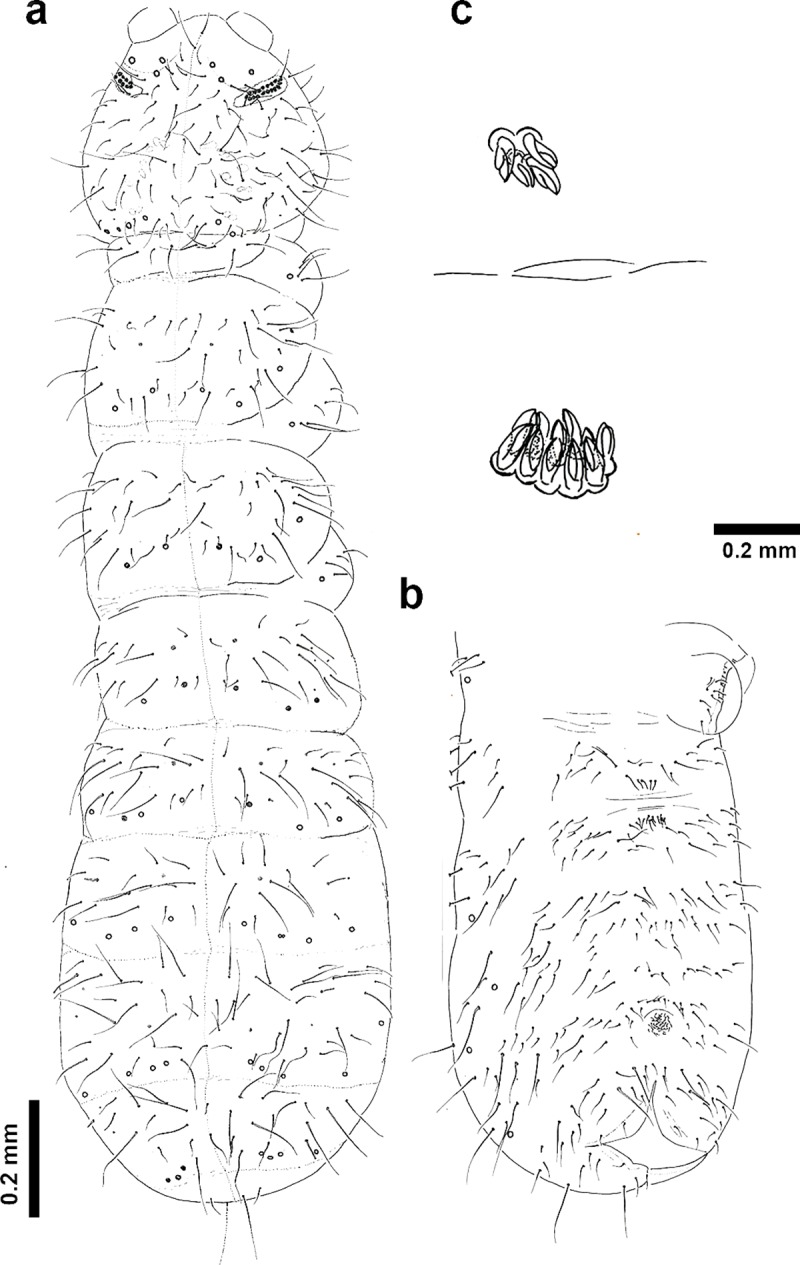
*Deuteraphorura muranensis* sp.nov. 4a –dorsal chaetotaxy of the body (axial line marked); 4b –ventral chaetotaxy of abdomen (subadult male); 4c –MVO.

**Fig 5:**
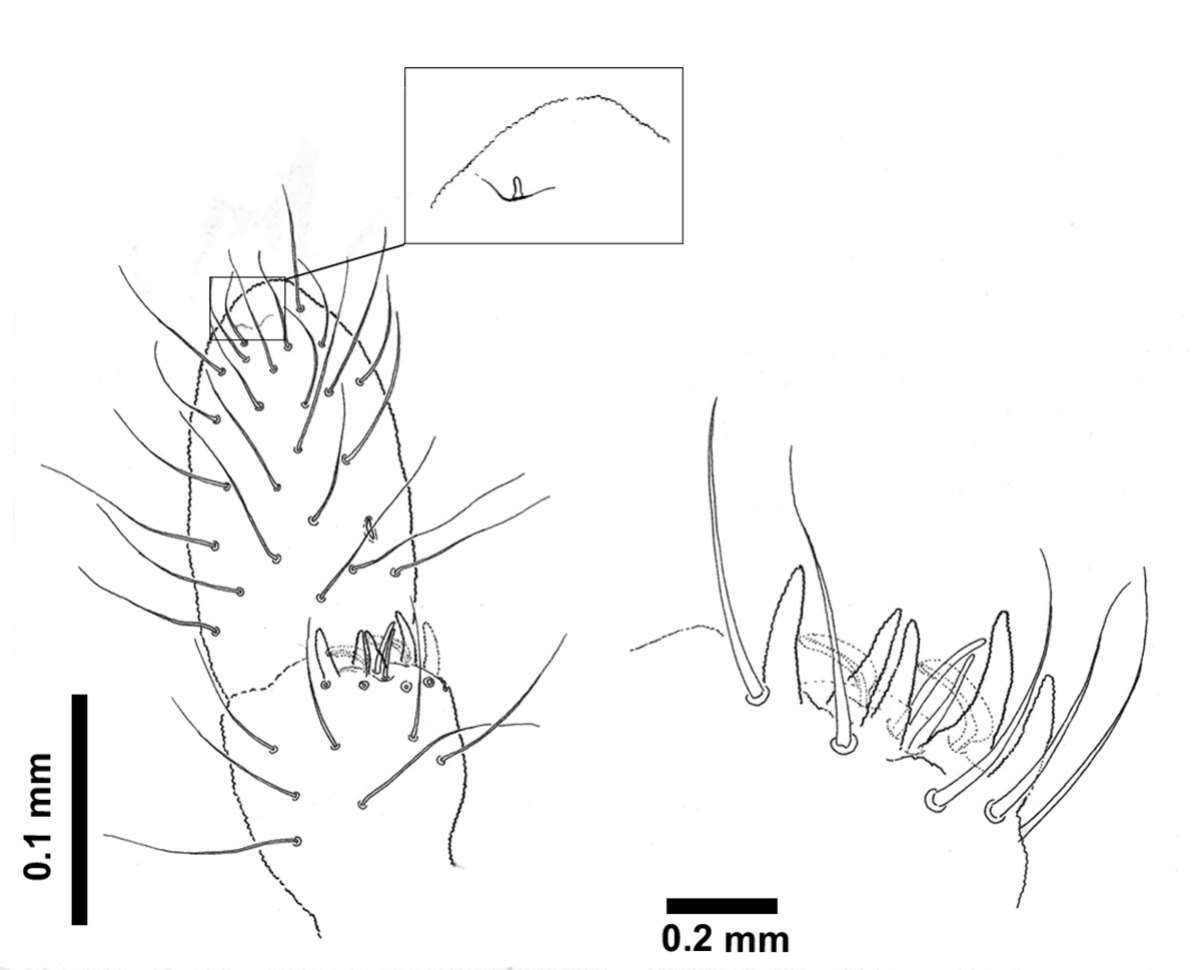
*Deuteraphorura muranensis* sp.nov.–dorso-lateral view of Ant. III-IV (left) with enlarged AOII (right) and apical organite (right above).

**Fig 6:**
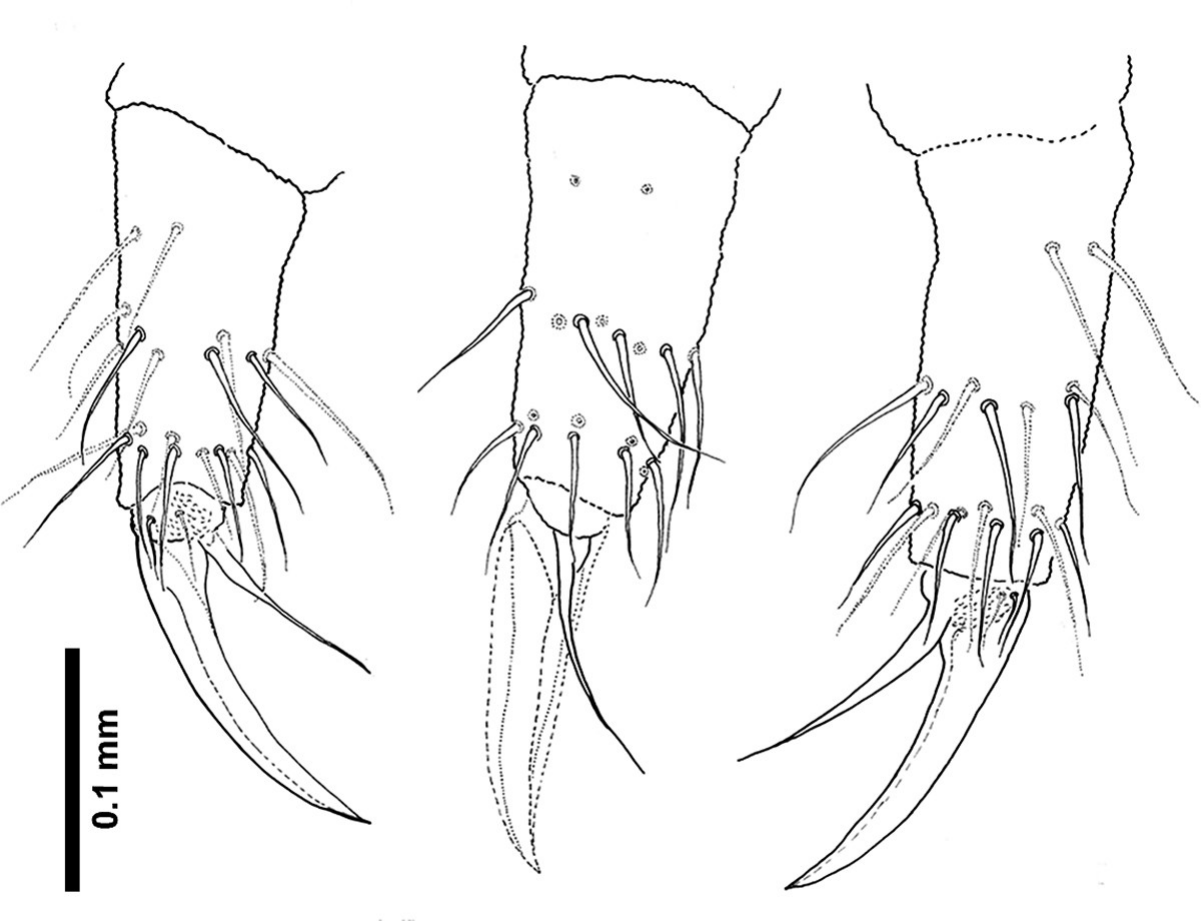
***Deuteraphorura muranensis* sp.nov.**–chaetotaxy of Tita I (left leg–lateral view), II (left leg–internal view) and III (right leg–lateral view), backside chaetae or bases of chaetae marked by dashed lines.

**Fig 7:**
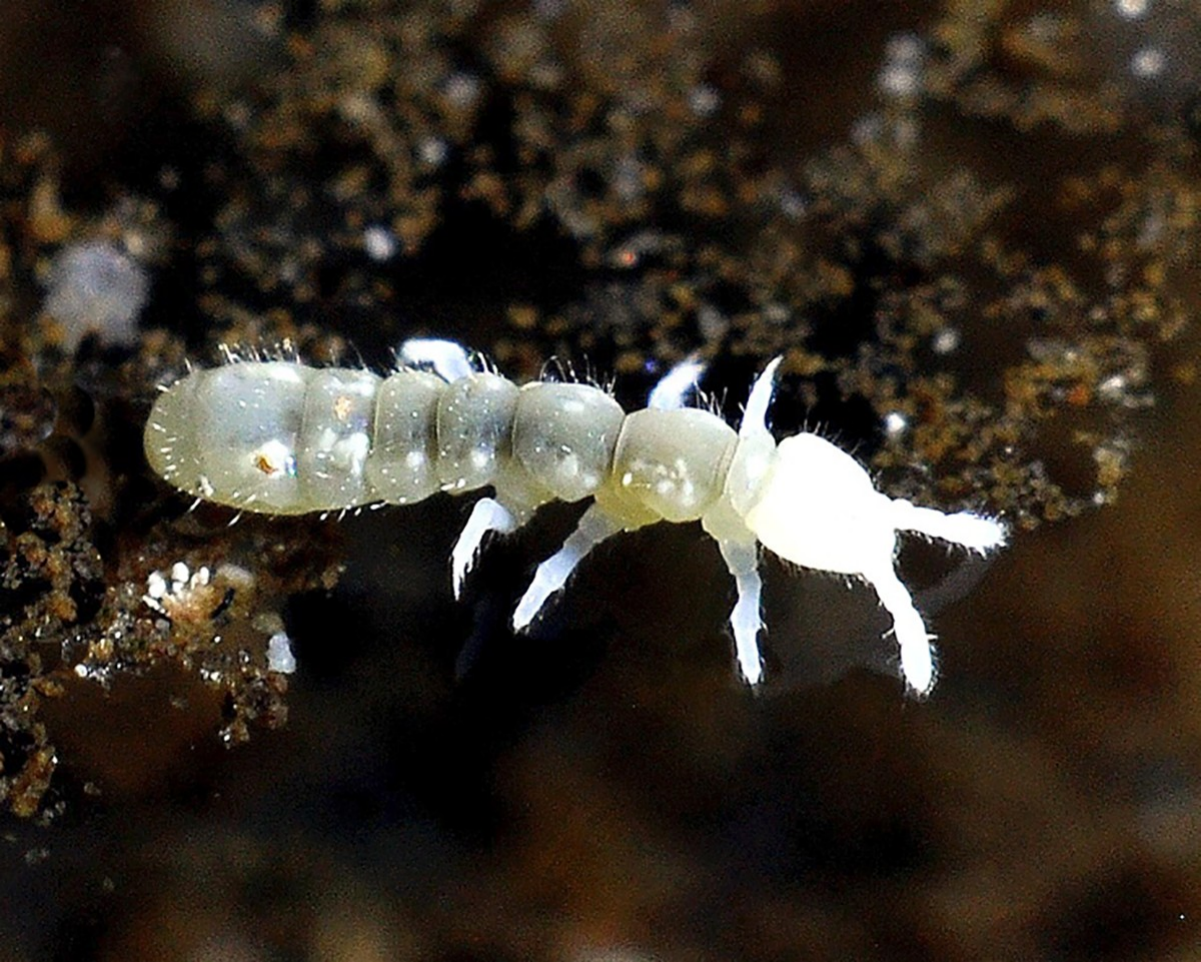
*Deuteraphorura muranensis* sp.nov.–specimen on the surface of a water pool, photo: Ľ. Kováč & A. Parimuchová.

**Fig 8:**
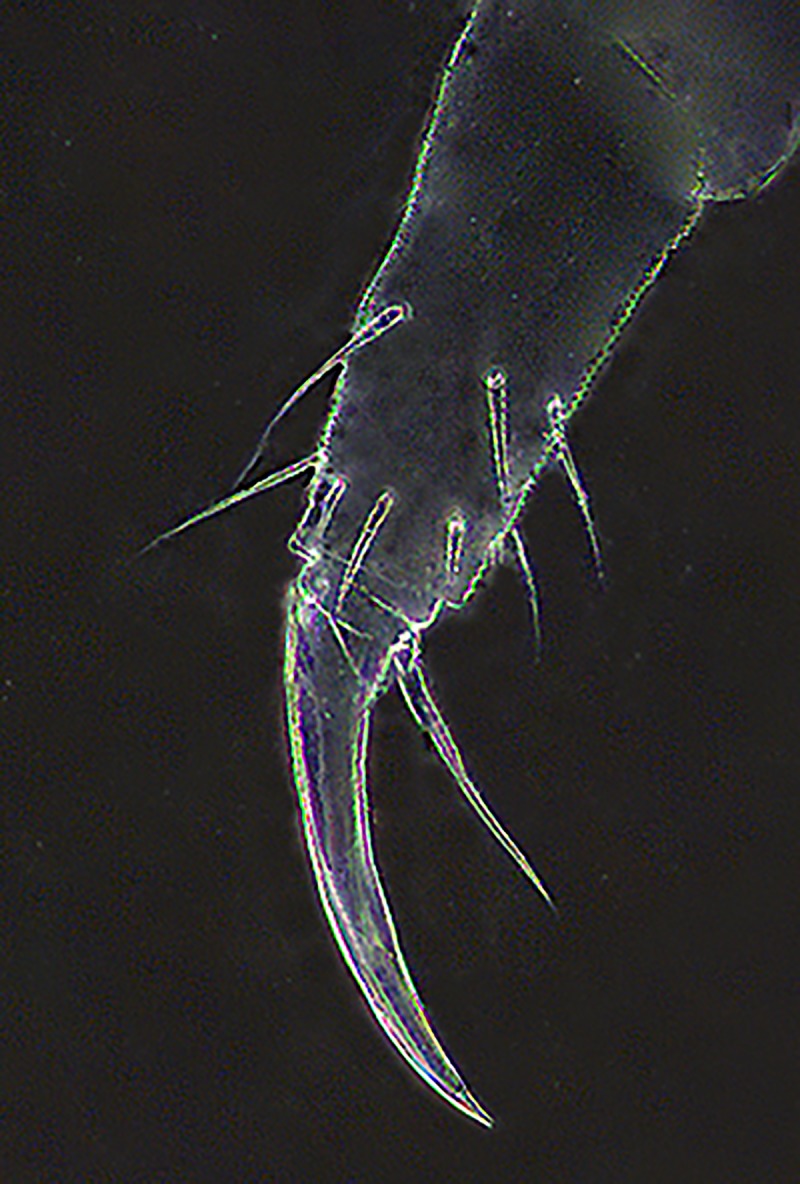
*Deuteraphorura muranensis* sp.nov.–tibiotarsus and foot complex of leg I.

**Fig 9:**
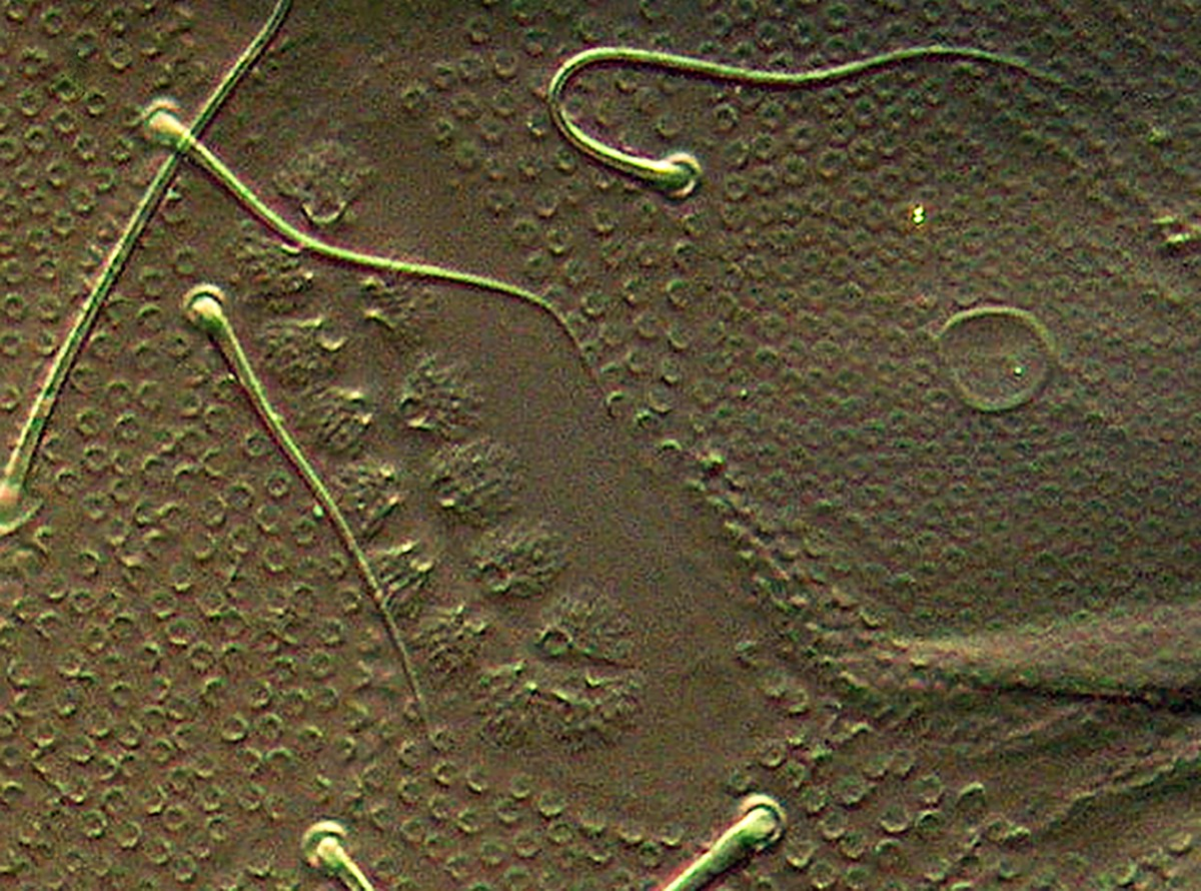
*Deuteraphorura muranensis* sp.nov.–PAO.

**Fig 10:**
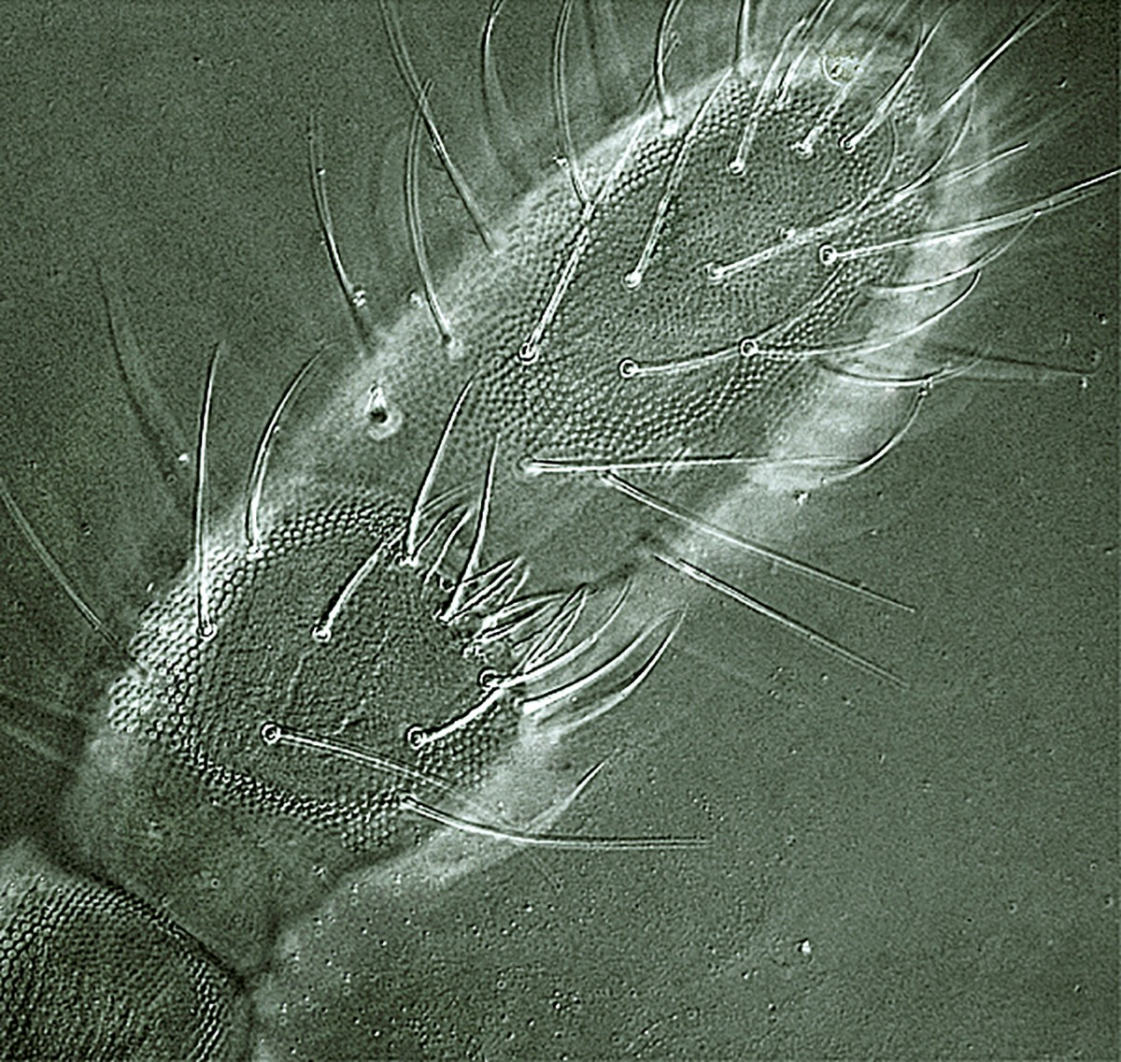
*Deuteraphorura muranensis* sp.nov.–dorso-lateral view of Ant. III-IV.

***Deuteraphorura muranensis*** Parimuchová & Kováč, **sp.nov. (Figs 4–10)**

urn:lsid:zoobank.org:pub: E74DDFF5-61CC-40E4-B0CE-4B9E9D3CC6B9

*Deuteraphorura* sp.2 [30]

### Material morphologically examined

*Holotype*. ♀ on slide, Slovakia: Muránska planina Plateau, Jelenia priepasť Abyss, Sieň pagody Hall, (No. 562–11), surface of the water pool, 2.ix.2011, leg. Ľ. Kováč

*Paratypes*. Ibid. Jelenia priepasť Abyss, Sieň pagody Hall, 3 ♂ (No. 562–11), water surface of a sinter pool, 2.ix.2011, leg. Ľ. Kováč; 1 ♂ (No. 90–18), water surface of a sinter pool, 24.iv.2018 leg. Ľ. Kováč and A. Parimuchová; Srnčia sieň Hall, 1 ♂ (No. 118–18), surface of the water puddle in clay sediment, 18.v.2018 leg. Ľ. Kováč and A. Parimuchová

*Other material*: 6 ♂ and 7 ♀on slides, Slovakia: Muránska Plateau: Bobačka Cave: internal part - Čapík, 1 ♂ and 1 ♀ (No. 174–00), hand collecting on bat guano, 5.x.2000, leg. P. Ľuptáčik and Ľ. Kováč; Chodba tušenia Passage, 1 ♀ (No. 271–11), hand collecting on water surface of a sinter pool, 9.v.2011, leg. Ľ. Kováč; Riečna chodba Passage, 1 ♀ (No. 207–00), hand collecting on organic bait, 9.xi.2000, leg. Ľ. Kováč.

**Description.** Body length 2.4–3.1 mm in females, 1.9–2.75 in males, shape cylindrical (Figs 4A and 7). Colour white, both alive and in ethyl alcohol. Cuticular granulation fine and uniform. Antennae as long as head, area antennalis relatively well marked. PAO with 14–22 compound vesicles (Fig 9). Ant. I with 8–9 chaetae in one row, Ant. II with 14–16 chaetae. AOIII with 5 papillae, 5 guard chaetae, 2 sensory rods almost as long as papillae, 2 rough sensory clubs and lateral ms. Lateral ms on Ant. IV placed basally at the level of second row of chaetae (Figs 5 and 10), apical organite simple in unprotected cavity. Labium of AB-type, with 6 proximal chaetae. Basomedian field with 4 chaetae, basolateral field with 5 chaetae. Maxillary outer lobe simple with 1 basal chaeta and 2 sublobal hairs.

Pso formula dorsally as 34/033/3-43-43-45-64 (Fig 4A) (sometimes 4 pso on Abd. I-II and 3 pso on Abd. III); ventrally as 11/000/0-100-10-10-1 (number of pseudocelli on Abd. sternum not stable, often with left-right asymmetry); head ventrally with 1 anterior and 1 postero-lateral pso. Psx weakly visible. Subcoxae 1 on legs I–III with 1,1,1 pso, respectively.

Dorsal body chaetae long, hair-like, often asymmetrically arranged. Th. I with 7–10 chaetae per half body. VT with 7–10 chaetae per half, basal chaetae absent. Abd. IV and V. with 1 and 2 axial (unpaired) chaetae respectively. Chaetae on Th. I–III sterna absent. Furca remnant with 2+2 thin chaetae in one row. MVO present in form of thickened leaf-like chaetae, 4 on Abd. II sternum and 10–13 on Abd. III sternum (Fig 4C). Subcoxae 1 of legs I–III with 4(5), 4–6 and 4(5) chaetae, subcoxae 2 with 0, 3(4) and 3(4) chaetae, coxae with 3, 9–12, 10–13, trochanters with 8–10, 8–10 and 9 chaetae, and femora with 15–17, 15–18 and 12–15 chaetae, respectively. Distal verticil of chaetae on tibiotarsi with 9 chaetae, as is typical for the genus. Tita I with 7+M chaetae in B row, 1 chaeta placed above and 1–2 chaetae in C row. Tita II with 7+M chaetae in B-row and 2 chaetae in C-row. Tita III with 6+M chaetae in B row and 2 chaetae in C row (Fig 6). Claw distinctly elongated without teeth, length/width ratio 3.0–3.7. Empodium with narrow basal lamella, tip of filament reaching two-thirds of claw length on legs I and about three-quarters on legs II and III (Fig 8).

**Biology.** *Deuteraphorura muranensis* **sp. nov** was collected in two caves of the Muránska planina Plateau, central Slovakia, almost exclusively on the water surface of sinter pools, but also on bat guano and organic baits in the deeper parts of the caves. The new species possesses distinctly elongated claws, one of the troglomorphic adaptations to cave life. These caves are type localities of another troglobiotic collembolan, an undescribed species of the genus *Pseudosinella*, likewise characteristic with conspicuously long claws.

**Etymology.** The species name is derived from the name of the closest village Muráň, with an old castle, and the karst region Muránska planina Plateau, where the caves inhabited by this species are situated.

### Remarks on the new species

***D*. *muranensis* sp. nov.** represents a well-defined species morphologically, genetically and geographically. It is the only representative of the genus *Deuteraphorura* with 4 pso on the hind margin of the head and the absence of pso on Th. I. Several species with 4 pso on the hind head margin have been described from caves, however, with pso on Th. I tergum. By the dorsal abdominal pso formula, the new species is most similar to congeners occupying caves in Spain (Table 2). Parapseudocellar and pseudoporal formulas have been used as determining characteristics in several Onychiuridae taxa, e.g. [22, 46, 47]; however, these characteristics are often only barely visible, e.g. [48], as in the case of *D*. *muranensis* **sp. nov**. In addition, the new species shows a high level of left/right asymmetry in dorsal chaetotaxy and the distribution of the ventral abdominal pso (Fig 4B).

**Table 2:** List of *Deuteraphorura* species with at least 5 pso on Abd. IV and 4 pso on Abd. V.

species	description	country	habitat	length (mm)	PAO ves	dorsal pso	ventral pso	subcoxae I
*D*. *ameskoana*	(Beruete, Arbea & Jordana, 2001)	Spain	caves	1.0–1.1	12–14	34/133/44464	3/011/3111	?
*D*. *aralarensis*	(Beruete, Arbea & Jordana, 2001)	Spain	caves	1.2	12–14	34/133/3-443-453-4	3/011/3211	?
*D*. *boneti*	(Beruete, Arbea & Jordana, 2001)	Spain	caves	1.2	12–14	34/133/3-44-53-454	3/011/41-21-22	?
*D*. *diaelleni*	(Neuherz & Nosek, 1976)	Austria	caves	up to 3.0	20–24	34/133/34463-4	1/000/0001	2,2,2
*D*. *doftana*	Weiner & Fiera, 2014	Romania	soil	1.2–1.4	11	33/033/33354	3/011/3212	2,2,2
*D*. *doneztebensis*	(Beruete, Arbea & Jordana, 2001)	Spain	caves	1.4–1.6	12–14	34/133/4445-64	3/011/3111	?
*D*. *galani*	(Beruete, Arbea & Jordana, 2001)	Spain	caves	1.0–1.2	12–14	33/133/3-443-454	3/011/4212	?
*D*. *harrobiensis*	(Beruete, Arbea & Jordana, 2001)	Spain	caves	1.1.– 1.3	12–15	33/133/3-44464	3/011/4111	?
*D*. *kratochvili*	(Nosek, 1963)	Slovakia	cave	1.5–2.2	15–17	33/033/33354	2-3/00-10-1/2212	2,2,2
*D*. *labainensis*	(Beruete, Arbea & Jordana, 2001)	Spain	caves	1.1.– 1.7	12–16	34/133/3-44464	3/011/3211	?
*D*. *leitzaensis*	(Beruete, Arbea & Jordana, 2001)	Spain	caves	1.3–1.4	12–14	34/233/3-44464-5	3/011/4112	?
*D*. *muranensis* sp. nov.	this paper	Slovakia	caves	2.4–3.1	14–22	34/033/3-43-43-45-64	2/000/0-100-10-1	1,1,1
*D*. *quadrisilvaria*	(Gisin, 1962)	Austria	cave	2.4–3.6	18	34/133/33354	3/011/3211	2,2,2
*D*. *rendsinae*	(Haybach, 1962)	Austria	soil	0.6–0.8	8–10	33/033/33354	2/000/1112	2,2,2
*D*. *simoni*	Arbea, 2017	Spain	soil	0.5–1	6–9	33/033/33354	2/000/0111	1,1,1
*D*. *trisilvaria*	(Gisin, 1962)	Austria	cave	1.6–2.4	18	33/133/33354	3/011/3211	2,2,2
*D*. *zalbidensis*	(Beruete, Arbea & Jordana, 2001)	Spain	caves	1.1	14–17	34/133/44464	3/011/4222	?

The claws of the new species (evaluated as average length/width ratio 3.3) are distinctly longer compared to other cave-dwelling congeners from the Western Carpathians–*D*. *schoenviszkyi* (Loksa, 1967) and *D*. *kratochvili*–with average length/width ratio 2.6 [30].

## Discussion

### Species delimitation and cryptic diversity in cave *Deuteraphorura* in the Western Carpathians

*D*. *kratochvili* represents the most widespread cave species, with a distribution range covering the Western Carpathian Mts, where the existence of two morphological forms, *D*. *kratochvili* and *D*. cf. *kratochvili*, with distribution limited to the adjacent karst areas was assumed [49, 30]. In our former study [30], morphological variability was found in all *Deuteraphorura* populations, but the studied characteristics were not population-specific; thus, we considered them all as belonging to the same species, *D*. *kratochvili*. However, there is another obligate cave congener in the Western Carpathian caves that has not been successfully collected for more than ten years. *Deuteraphorura schoenviszkyi* (Loksa, 1967) is endemic to the Slovak and Aggtelek Karst and represents a morphologically well-defined species with distinctly elongated claws [49]. Unfortunately, no molecular analysis has yet been done, since occurrence is very rare in the caves of the area.

An integrative approach has been repeatedly highlighted as the best possible future of taxonomy, employing morphological and molecular attributes as well as attributes of the extended phenotype, behaviour and ecology [15, 19, 22, 23, 24]. We applied morphological, molecular and geographical approaches to resolve the taxonomic status of the cave *Deuteraphorura* representatives occurring in the Western Carpathians. Four *D*. *kratochvili* populations and one population of the new species were used in this integrative study. Although *D*. *kratochvili* occupies most Western Carpathian caves, the sampling of a sufficient number of individuals for both molecular and morphology analyses was highly demanding in most of them due to difficult access to individual cave sites and the rather dispersed distribution of the individuals in caves in general.

In the present study, groups of specimens defined by ABGD and PTP analyses respectively, corresponded to particular caves (populations). However, an additional separation was indicated within two populations, namely Jelenia priepasť Abyss and Demänovská Ice Cave. Similarly, relatively large intraspecific distances (4% or greater) have been reported within several collembolan species, e.g. [16, 17, 24, 26, 27]. Interpopulation divergence in *D*. *kratochvili* was higher than between the populations of *P*. *janosik* Weiner, 1990, from several Western Carpathian caves [34], suggesting longer isolation and evolution of *D*. *kratochvili*, thus probably representing an “old troglobiont”, i.e. a troglobiont that is a descendant of an old phyletic lineage [4].

Since reliable species boundaries are difficult to delimit by genetic distance alone, and morphological differences are indistinct in *D*. *kratochvili*, we used a geographical criterion, which proved to be an efficient delimitation method for onychiurids [22]. The results of both molecular analyses and the geographical distance between localities suggested the existence of genetically isolated forms of *D*. *kratochvili* with positively correlated geographical and genetic distances. A similar correlation has been documented in several cave collembolan populations [21, 27, 34] and coincides with the intensity of gene flow. In Collembola, gene flow was confirmed across large geographical distances, but in contrast, also a minimal sharing of haplotypes, and thus reproductive isolation may be demonstrated between adjacent or even sympatric species that are indistinct morphologically [17, 50]. In geographically distant and physically isolated populations, some intra-specific genetic distance has been reported, even though only 3–4% [27, 34]. The intraspecific distance in *D*. *kratochvili* (11%) was higher than minimal interspecific divergence in Chinese *Protaphorura* documented in two species separated by more than 1000 km [22]. Based on these results, it is clear that *D*. *kratochvili* represents a complex of several species. The absence of reliable morphological characteristics and/or the high level of their variability were the reasons why traditional taxonomic methods failed to reveal these cryptic species.

An increasing trend in the number of collembolan species is obvious, since molecular methods have become an integral part of taxonomy. The diversity of Collembola is greatly underestimated owing to the prevalence of cryptic species [51]. Whereas 14% was adopted as the threshold for species delimitation [42], it has not been strictly defined. Ubiquist species with large distribution ranges, including several more or less geographically isolated populations, have a high intraspecific (interlineage) variability reaching more than 20%, and particular lineages may represent cryptic species [25, 26]. Along with cryptic (morphologically uniform) species with surprisingly high interspecific distances, morphologically, geographically and/or ecologically well-defined species with low interspecific distance have also been observed [29]. In some species previously identified as cryptic taxa by molecular distance, additional morphological differences were found after subsequent more thorough, detailed morphological re-examination of individuals [19]. Finally, if only genetic distance is applied for species delimitation, some species could be neglected, as low divergence between them would be assigned as intraspecific variability.

Generally, without thorough study of morphology and the use of geographical distance data species delimitation may be biased. Due to the lack of any morphological differences and minor interspecific (JPA populations = *D*. *muranensis* vs. OAC populations = *D*. *kratochvili)*, major intraspecific (*D*. *kratochvili*) molecular divergence as well as interspecific distance smaller than intraspecific, we consider *D*. *kratochvili* to be a complex of cryptic species in which long-term isolation in individual karst systems likely led to gene-flow cut-off.

### Troglomorphy of cave Collembola at the northernmost distributional border in Europe

In Europe, troglobiont diversity increases towards the southern mountains (Pyrenees, Alps, Dinaric Mts), reaching a maximum in so-called “hot-spot” areas [3]. The present study contributes to the recent findings that the Western Carpathians are the northernmost limit of the occurrence of the troglomorphic taxa in Europe, inhabited by several obligate cave Collembola species [4, 30, 34, 49, 52, 53]. The distribution pattern of “hot-spot” areas is basically associated with the productivity of surface ecosystems, spatial heterogeneity and historical conditions covering the effect of the past climatic and paleogeographic events [54]. The presence of such a hotspot reflects not only the local diversity of troglobionts but may also be associated with the high level of troglomorphy in these obligate subterranean forms. A heterogeneous community of highly troglomorphic collembolan taxa of the families Tomoceridae, Entomobryidae, Arrhopalitidae and Neelidae may occupy the caves of a “hot-spot” area [55, 56, 57, 58, 59], while the level of troglomorphy is only moderate in troglobionts at the borders of the distributional range [4, 49, 53]. Morphological adaptations seem to be the most distinct in Entomobryidae, while basically inconspicuous in Onychiurinae, a subfamily which is “uniform” in general morphology and ecology. Moreover, regressive morphological changes, such as loss of pigmentation and eyes characteristic in edaphic taxa, could not be assigned as troglomorphisms. One morphological manifestation of troglomorphy in Collembola is elongation of body appendages [7, 60]. In Onychiuridae, elongation of morphological structures in troglobiotic representatives is most distinct in the foot complex. Generally, the ratios claw length/width, claw length/Tita length or claw length/body length have been used to estimate the level of troglomorphy, e.g. [12, 30, 34, 59]. Despite the fact that the subfamily Onychiurinae is extremely edaphic with several eutroglophile forms, only three highly troglomorphic species are known: two species of *Ongulonychiurus* and *Troglaphorura gladiator*. Whereas both *Ongulonychiurus* are distributed within hot-spot areas (Picos d´Europa, Spain and Biokovo Mts, Croatia), *Troglaphorura* was discovered in the Western Caucasus, representing indeed an important “hot-spot” area of the subterranean fauna with a great potential for further important discoveries of the highly specialized subterranean invertebrates.

Endemic distribution of particular species in a “hot-spot” area is not indeed a “guarantee” of a high level of troglomorphy in the local taxa, as shown in several *Deuteraphorura* species from caves of Navarra (Spain) [61]. *Absolonia gigantea* (Absolon, 1901), occupying caves of the Balkan Peninsula, displays the same level of troglomorphy as *Protaphorura janosik* Weiner, 1990 [34] and *P*. *cykini* [62] from the Western Carpathians in Slovakia and the Baikal territory in Siberia, respectively, achieving a larger body size compared to other congeners. Moreover, recent discoveries of new troglomorphic taxa in previously “biospeleologically neglected” localities [12] document that the area with a high degree of troglomorphy in Collembola is larger in Europe than was defined by Culver et al. [3]. On the other hand, discoveries of troglomorphic taxa and the diversity of troglobionts in this group also depend, among other things, on the number of caves in the individual region, the local rate of biospeleological exploration and the sampling effort.

The highly morphologically adapted subterranean species of the genus *Deuteraphorura* described in this paper was found in two caves of the Muránska planina Plateau, a small karst area in central part of the Western Carpathians (213.2 km^2^ in area) with ~500 caves. We are not able to describe the possible governing factors that led to the evolution and endemic distribution of this highly troglomorphic species in this area. In fact, Christiansen & Culver [63] noted the relation between the level of troglomorphy and distribution-range size in Collembola, i.e. that increasing troglomorphy decreases their ability to disperse. Similarly, *D*. *schoenviszkyi* has endemic distribution and displays a higher level of troglomorphy than *D*. *kratochvili*, which has greater distribution range [30, 49]. In addition, increasing troglomorphy indicates early (pre-glacial) times of first cave colonization [63]. Of course, this remains to be tested using a phylogenetic approach based on a broader molecular dataset originating from multiple *Deuteraphorura* populations across the Western Carpathian caves.

## Abbreviations

Ant: antennal segment
Th: thoracic tergum
Abd: abdominal tergum
VT: ventral tube
Tita: tibiotarsus
AOIII: antennal organ of the third antennal segment
ms: microsensillum
MVO: male ventral organ
PAO: postantennal organ
pso: pseudocellus
psx: parapseudocellus
IBE FS UPJS: Institute of Biology and Ecology, Faculty of Science, P. J. Šafárik University, Košice, Slovakia
